# Functional role of microRNA in the regulation of biotic and abiotic stress in agronomic plants

**DOI:** 10.3389/fgene.2023.1272446

**Published:** 2023-10-10

**Authors:** Ramkumar Samynathan, Baskar Venkidasamy, Ashokraj Shanmugam, Sathishkumar Ramalingam, Muthu Thiruvengadam

**Affiliations:** ^1^ Department of Oral and Maxillofacial Surgery, Saveetha Dental College and Hospitals, Saveetha Institute of Medical and Technical Sciences, Saveetha University, Chennai, Tamil Nadu, India; ^2^ Plant Physiology and Biotechnology Division, UPASI Tea Research Foundation, Coimbatore, Tamil Nadu, India; ^3^ Plant Genetic Engineering Lab, Department of Biotechnology, Bharathiar University, Coimbatore, Tamil Nadu, India; ^4^ Department of Crop Science, College of Sanghuh Life Science, Konkuk University, Seoul, Republic of Korea

**Keywords:** miRNA, stress response regulation, biotic and abiotic stress, gene expression, agronomic traits

## Abstract

The increasing demand for food is the result of an increasing population. It is crucial to enhance crop yield for sustainable production. Recently, microRNAs (miRNAs) have gained importance because of their involvement in crop productivity by regulating gene transcription in numerous biological processes, such as growth, development and abiotic and biotic stresses. miRNAs are small, non-coding RNA involved in numerous other biological functions in a plant that range from genomic integrity, metabolism, growth, and development to environmental stress response, which collectively influence the agronomic traits of the crop species. Additionally, miRNA families associated with various agronomic properties are conserved across diverse plant species. The miRNA adaptive responses enhance the plants to survive environmental stresses, such as drought, salinity, cold, and heat conditions, as well as biotic stresses, such as pathogens and insect pests. Thus, understanding the detailed mechanism of the potential response of miRNAs during stress response is necessary to promote the agronomic traits of crops. In this review, we updated the details of the functional aspects of miRNAs as potential regulators of various stress-related responses in agronomic plants.

## Introduction

MicroRNAs (miRNAs) are small, non-coding, single-stranded RNAs, which are 18–24 nucleotides in length. miRNAs play a crucial role in numerous biological processes in plants (Bartel and Bartel, 2003) and animals (Stefani and Slack, 2008). It has been shown that miRNAs are responsible for morphogenesis, growth and development, hormone secretion, signal transduction, and environmental stress responses (Liu et al., 2017), and the post-transcriptional regulation of genes is closely associated with agronomic traits (Zheng and Qu, 2015). miRNAs represent approximately 1%–2% of the total genes of an individual plant (Zhang et al., 2008). High-throughput sequencing is an efficient technique for identifying plant miRNAs (Zhu et al., 2010). Several reports have stated that changes in the external environment of plants could alter the miRNA profile, which regulates various stress responses in plants (Lu et al., 2011; Yang et al., 2013; Bej and Basak, 2014). Recently, miRNA identification has emerged as an important target. Deep sequencing technologies have been used to identify and understand the species-specific functions of miRNAs and their target genes (Amanda et al., 2013). The miRNA database (miRBase) v.21. maintained 7,385 plant miRNAs. Among these, 337 miRNAs were from Arabidopsis, 713 from rice, 321 from maize, and 241 and 61 from sorghum and barley, respectively. In plant species, sixteen miRNA families with a central function in stress response have been reported (Chen et al., 2016). Additionally, MYB domain protein transcription factors (TFs), a major TF family present in plants, control the miRNA-regulated aspects of plant growth, metabolism, and stress response. miRNAs provide an adaptive mechanism to plants for survival under environmental stress, such as drought, salinity, cold, heat, and pathogenic conditions. Moreover, the potential mechanism of miRNAs in the stress response is necessary to increase the yield of agronomically important crop varieties (Djami-Tchatchou et al., 2017; Zhang et al., 2022). In this regard, this review features the biogenesis and mechanism of regulation in miRNAs and various functional aspects of miRNAs in governing the environmental stress responses in economically viable agronomic plants. This review presents updated information on the functional characteristics of miRNAs as potential regulators of stress-related responses in agronomic plants.

## Biogenesis and processing of miRNAs

**FIGURE 1:**
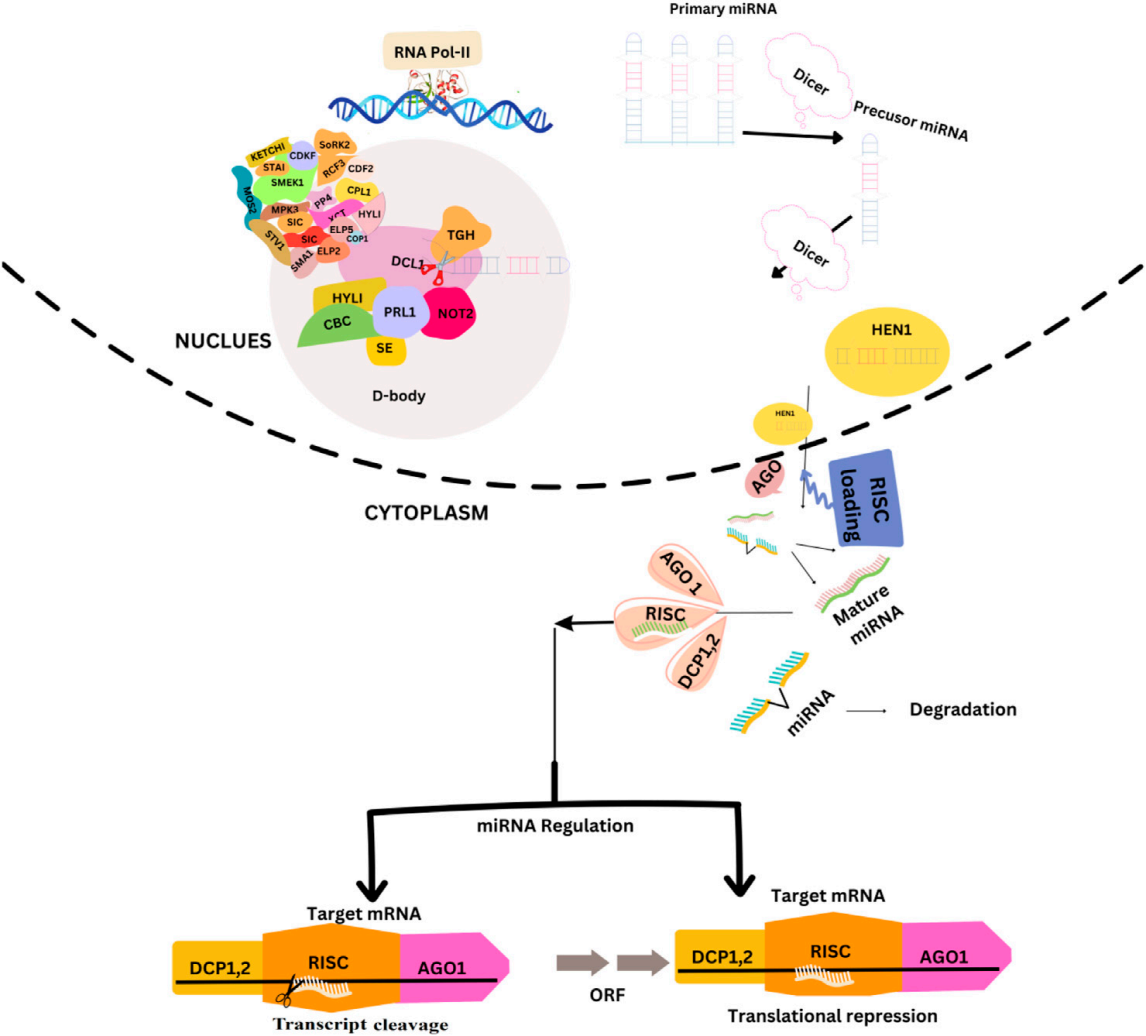
The regulation of microRNA (miRNA) biosynthesis and its functional mechanisms in plants. miRNAs are initially transcribed as primary miRNAs (pri-miRNAs) by the enzyme DNA-dependent RNA Polymerase II (Pol II). Subsequently, these pri-miRNAs undergo processing to form precursor miRNAs (pre-miRNAs) by the action of a protein complex known as D-body. After undergoing processing, the miRNAs in their double-stranded form are subjected to methylation by the enzyme HEN1. Subsequently, they are transported from the nucleus to the cytoplasm with the assistance of HST. The mature miRNA, that is, produced can downregulate gene expression by integrating into AGO1, which subsequently associates with other proteins to assemble the RNA-induced silencing complex (RISC). There are two distinct mechanisms through which RISC (RNA-induced silencing complex) exerts its effects: transcriptional cleavage and translation suppression.

The miRNA genes are mainly situated in the intergenic region (IGR) but are also found in the sense or antisense orientation within the introns. RNA polymerase II transcribes the miRNA genes in the nucleus to generate pre-miRNAs. The pre-miRNA consists of long hairpin structures with nucleotide lengths ranging from 70 to hundreds of bases. The pre-miRNA undergoes a sequence of processes leading to the formation of a solitary hairpin structure, which is then cleaved by Dicer-like (DCL) enzymes to generate a mature miRNA (Axtell et al., 2011). First, DCL1 is linked to chromatin regions of the MIR gene (Fang et al., 2015). Secondly, many regulatory proteins regulating miRNA transcription (such as CDC5, NOT2, and ELP2) have been found to interact with processing machinery proteins (Wang et al., 2019). Plant miRNAs perfectly or near-perfectly match their mRNA targets and mediate the silencing or direct cleavage of complementary target mRNAs (Jones et al., 2006). To recognize the target mRNA, the miRNA is transported to the cytosome by HASTY (HST) and combined with RNA-induced silencing complex (RISC) recognizing the target mRNA and leading to the cleavage or repression of translation (Park et al., 2005). In addition, some miRNAs are located in host intron genes known as mitrons. Mitrons are widely distributed in animals; however, only a few have been identified in rice and Arabidopsis (Meng and Shao, 2012). However, the precursor of mitrons is located in the intron of an unknown protein-coding gene. The regulation of microRNA biosynthesis and its functional mechanisms in plants are shown in Figure 1.

## MiRNA conservation and gene regulation

miRNA families are grouped into conserved and non-conserved miRNAs based on their evolutionary conservation and diversification. Researchers have identified conserved and novel miRNA sequences from plant tissues, such as leaves, stems, calluses, and flowers, using deep sequencing techniques. Several studies have demonstrated the conservation of miRNAs across several plant taxa, including gymnosperms, dicots, eudicots, and mosses (Cuperus et al., 2011; Sun, 2012). Evolutionarily conserved miRNAs were highly expressed. In contrast, non-conserved miRNAs are expressed at low levels under specific conditions and exist in limited species (Taylor et al., 2014). The significance of miRNAs in plants was first identified from conserved miRNA sequences, such as miR319 and miR172, which are involved in the morphogenesis of both leaves and flowers (Palatnik et al., 2003). miRNAs, such as miR156, miR160, miR165, miR166, miR167, miR319, miR390, miR395, and miR408, are conserved among terrestrial plants and non-flowering moss (Taylor et al., 2014). Moreover, miR156 to miR408 are conserved miRNAs that are common between gymnosperms and angiosperms (Cuperus et al., 2011; Taylor et al., 2014). Bonnet et al. (2004) analyzed the conservation of miRNAs between the genomes of Arabidopsis and *Oryza sativa* and they identified 91 potential miRNAs, among those 58 miRNAs were relatively matched.

Non-conserved miRNAs are unique in specific species that possess immense targets, such as active enzymes and proteins involved in adaptation to diverse conditions. For example, miR2275 in rice, miR173 in Arabidopsis, and miR482 in tomato are non-conserved miRNAs (Shivaprasad et al., 2012). Micro RNA regulates gene expression through different mechanisms. The miRNA specifically recognizes the target mRNA gene which possesses complementarities to the miRNA followed by the activation or inhibition of target genes (Sun, 2012). Typically, miRNAs bind to the 3′- untranslated region (3′-UTR) of target mRNAs, followed by destabilizing and translational silencing of the mRNA, which leads to the inhibition of protein production. Moreover, miRNAs have specific functions in the cytoplasm and the nucleus. In the cytoplasm, the miRNA-induced silencing complex (miRISC) regulates the fate of miRNAs by inhibiting translation or promoting mRNA degradation. However, in the nucleus, miRNA regulation is independent of RISC. Before the export of miRNAs to the cytoplasm, they bind with some target mRNAs in a pre-silenced state (Cannell et al., 2008). Budak and Akpinar (2015) reported that “Intergenic” and “intronic” miRNAs are the two different types of miRNAs. Intronic miRNAs are processed from the introns of their host transcripts, whereas intergenic miRNAs are situated between two protein-coding genes and are produced as separate units by DNA-dependent RNA Polymerase II (Pol II).

The canonical biogenesis pathway is the major pathway for miRNA processing and consists of an RNA binding protein, DiGeorge Syndrome Critical Region 8 (DGCR8), and a ribonuclease III enzyme Drosha, which transcribes pri-miRNAs from their genes and then processes them into premiRNAs. Plant MIRs, such as glycine-rich RNA-binding protein 7 (GRP7), STA1, and ILP1/NTR1, undergo splicing and alternative splicing, which can impede pri-miRNA processing. GRP7 is a hnRNP-like glycine-rich RNA-binding protein that enhances primiRNA splicing *in vivo* (Köster et al., 2014). The AGO protein serves as both an effector and a crucial factor in maintaining miRNA stability. The proteins HESO1, URT1, SDN1, and ATRM2 have been observed to interact with AGO1 and are involved in the regulation of AGO1-bound miRNAs or unmethylated miRNA/miRNA duplexes, as reported by Chen et al. (2018) and Wang et al. (2018).

Plants must maintain intracellular miRNA homeostasis in order to adapt to environmental and developmental changes. MicroRNA stability control, in addition to miRNA synthesis, is important for the regulation of miRNA homeostasis. MicroRNAs have significant regulatory functions in organisms through their ability to target certain messenger RNAs (mRNAs) for cleavage or translational repression. They exert influence over a wide range of biological processes, including leaf, root, and flower development, as well as grain filling (Bian et al., 2012). During the initial stages of grain development, there is a notable upregulation of miRNAs that target genes associated with cell differentiation, auxin-activated signalling, and transcription. This upregulation potentially contributes to the regulation of grain size. Subsequently, in the middle and later stages of development, miRNAs are found in abundance and target genes involved in carbohydrate and nitrogen metabolism, transport, and kinase activity. This abundance of miRNAs may play a role in grain-filling processes (Hou et al., 2020).

## Functional roles of miRNAs in agronomic plants

### Rice

**FIGURE 2:**
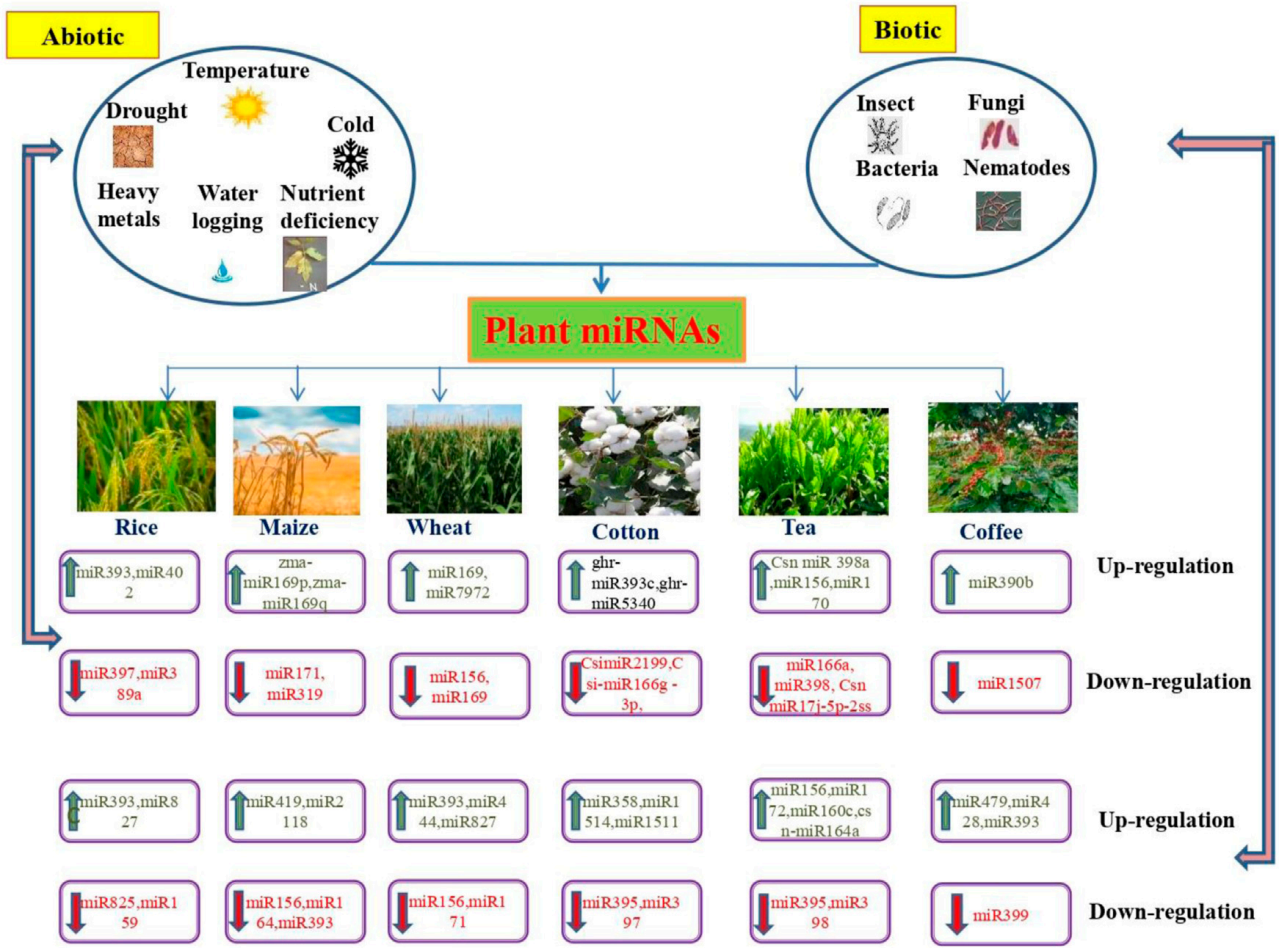
Functional roles of microRNAs (miRNAs) in abiotic and biotic stress response in agronomically important plants.

Rice (*O. sativa* L) is the most significant crop which feeds about two billion people worldwide. Moreover, the degree of rice production influences the country’s economic status. Environmental factors, including salinity, drought, temperature, and nutrient starvation affect the yield of rice crops. The recognition of miRNAs in rice has been rapidly improved by advances in sequencing technologies (Jeong et al., 2011; Yang et al., 2014). miRBase v.21 maintains 592 miRNA sequences that generate 713 mature miRNAs identified in rice (Kozomara and Griffiths, 2014). In rice, miRNAs can determine the yield, quality, and biotic and abiotic stress tolerance (Figure 2). Furthermore, miRNAs, small interfering RNAs (siRNAs) are also involved in stress responses (Bostock, 2005) and rice trait improvement (Ding et al., 2011; Yan et al., 2011; Wei et al., 2014). Salinity is a crucial abiotic stress that contributes to a 5% loss of crops worldwide (Gupta and Huang, 2014), affecting plant growth through irregular cell division, defective metabolism, and necrosis, which leads to cell death (Ayala and Alcaraz, 2010). The root is the primary organ that responds to soil-related stressors. However, post-stress conditions were reflected in the leaves. Primarily, miRNAs from leaf tissues are usually used to recognize the genome related to salinity stress in rice (Walia et al., 2005; Kumar et al., 2013). osa-miR169g and osa-miR169o were upregulated in rice during salinity stress. Further, osa-miR396c (Ding et al., 2009), and osa-miR393a (Gao et al., 2011; Ganie et al., 2016), osa-miR166 (Ding et al., 2009) are salinity-responsive miRNAs which were identified in rice (Table 1).

**TABLE 1:** Functional aspects of miRNA and their target genes in biotic and abiotic stress response in agronomic plants.

Plants	miRNA –Up-regulation	Functions	Target genes	References
Rice (*Oryza sativa*)	miR159, miR169, miR171, miR319, miR395, miR474, miR845, miR851, miR854, miR896, miR901, miR903, miR1026 and miR1125	Drought response	*MYB, HAP2, SCL, MYB, APS, TCP, SO2* transporter	Zhou et al. (2010)
	miR169g, miR171a and miR393	Induced in response to dehydration	CBF/DREB	Jian et al. (2016); Li et al. (2010)
	miR169, miR397, miR528, miR827, miR1425, miR319a.2 and miR408-5p	H_2_O_2_-responsive		
	miR156k, miR166m, miR166k, miR167a/b/c, miR168b, miR169e, miR169f, miR169h, miR171a, miR 1884b, miR319a/b, miR444, miR535, miR1320, miR1435, miR 1850, miR 1868, and miR1876	Cold stress	Cd-responsive miRNAs encode TFs	Lv et al. (2010)
	miR319a/b	Cold tolerance	*DREB1A/B/C*, *DREB2A*, *TPP1/2*	Yang et al. (2014)
	miR528, and miR827	H_2_O_2_-oxidative stress	Protein with F-box and SPX domain	Li et al. (2010)
	miR2118	Photoperiod-sensitive male sterility	*PMS1T*	Ta et al. (2016)
	miR168	Flower and yield improvement	*AGO1*	Wang et al. (2021)
	OsmiR535	Leaf senescence alteration	*OsSPL19*	Yue et al. (2020)
*Oryza glaberrima* and *Oryza barthii*	osa-miR528 and osa-miR408	Cd concentration of Cd in panicle node and grain	*OsMYB5P, OsbZIP18, OsERF141, OsSnRK1*, and *OsAAE3*	Liu et al. (2020)
Barley (*Hordeum vulgare*	miR166a, miR166′b, miR166c	Vascular differentiation, development of leaf and root formation	HD -Zip protein 8	Yadav et al. (2021)
	miR397a	Plant and flowering development	laccase precursor protein; transporter family protein	Huang et al. (2020)
	miR164	Lateral root and shoot development	ARF TF	Deng et al. (2015)
Sorghum (*Sorghum bicolor* (L.) Moench)	microRNAs include miR156a, miR156b, miR156c-f, miR156g-h, miR529, novel-sbi-miR-119, novel-sbi-miR-383, novel-sbi-miR-329, and a novel-sbi-miR-350	Response to drought stress	SBP gene	Katiyar et al. (2015)
	miR529		Protease and ubiquitin-related genes	
	miR398		Cu/Zn SOD, selenium-binding protein, and cytochrome C	
*Pennisetum glaucum* (L.) R. Br. (Pearl millet)	miR155	Response to drought stress	Cu-Zn superoxide dismutase, *eIF-4A*	Chakraborty et al. (2020)
	miR399, KN1		*NCED1, LOV,* aspartate aminotransferase	
*Setariaitalica* (L.) P. Beauv. (Foxtail millet)	miR160	Response to drought stress	*WRKY* and *ARF*	
	miR165 Carboxylesterase		*WRKY, ARF, NDR1/HIN1*	
Maize (*Zea mays* L.)	miR164, and miR160	Regulation of ABA metabolism	MYB, NAC, and ARF	Liu et al. (2019)
	miR393	Enhancing water potential and Aux/IAA protein degradation	*TIR1*	Seeve et al. (2019)
	miR172a, and miR160f-5p	Precondition of aerobic respiration	*ERTF RAP2-7, ISOFORM X2*, and *ARF8*	Azahar et al. (2020)
	miR162, miR168, miR395 and miR474	Salinity stress	SBP protein domain 6	Ding et al. (2009)
	miR166	Enhancing the pollination	HD zip transcription factor	Mica et al. (2006)
	miR169	Seed development	CCAAT- binding factor, HAP-2-like protein	Mica et al. (2006)
	microRNAs such as miR437, miR854, iR1128, miR1132, miR1133, miR1320, miR1435, miR1436, miR1439, miR 1884, and miR2102	Regulation of metabolic pathways	Maize transposon-related repeats	Liu et al. (2014)
Wheat (*Triticum turgidum* L.)	miR399b, miR393c, and ttu-novel-61	Enchaining the nitrogen stress during the grain filling	*PHO2, AFB2, CCAAT-TF*	Zuluaga et al. (2017)
	miR1139	Pi starvation enchantment	*TaMIR1139*	Liu et al. (2018)
	miR 1867, miR896, miR398, miR528, miR474, miR1450, miR396, miR 1881, miR894, and miR156	Drought- resistant wild emmer	DUF1242, plantacyanin, copper superoxide dismutase, POD, protein phosphatase PP2A-4 & SQUAMOSA promoter binding protein-like (SPL)	Kantar et al. (2011)
	miR393, miR444and miR827	Powdery mildew disease	Auxin signalling pathway genes	Xin et al. (2010)
	m0868_3p, m0874_3p, and m0220_3p	Embryogenic callus formation	Zinc finger MYB and SPL proteins	Chu et al. (2016)
	miR396a, miR444c.1,172a, miR393, miR167d, miR167c) and tasiRNA	Cold stress	ARF	Tang et al. (2012)
	miR156, miR159, miR160, miR166, miR168, miR169, miR393 and miR827	Heat stress	*ARF10*, *ARF16*, and *ARF17*, *ARF3* and *ARF4*	Xin et al. (2010)
	miR156	Juvenile-to-adult transition	BP-box transcription factors	Wang et al. (2017)

Other miRNAs such as miR156, miR157 and miR172, targeted SPB-like proteins (SPLs) and APETELA 2 (AP2), respectively, and also controlled salinity tolerance. miR393 is also involved in the drought response in rice, which is overexpressed under drought conditions and reduces both drought and salt tolerance in rice (Xia et al., 2012). miR169 helps control plant growth during salinity stress by altering the elongation of plant cells and carbohydrate metabolism by targeting the nuclear factor Y subunit (NF-Y) (Zhao et al., 2009). MiR164 regulates miRNA-mediated cleavage of NAC domain-containing protein TFs (NAC TFs). miR159 regulates MYB TFs responsible for flowering of rice plants under saline stress. However, the overexpression of MYBs leads to delayed flowering. Moreover, some salt-responsive miRNAs are responsible for protein turnover. For example, the genes responsible for the ubiquitin-proteasome pathway F box, Cullin-1, are an interesting new gene (RING) finger, and 4-coumarate-CoA ligase 1 protein takes part in the response to salinity stress (Sadanandomet et al., 2012; Singh et al., 2015). Mondal et al. (2015) reported that osa-miR164e repression by salt treatment results in elevated expression of ubiquitin-specific protease (UBP) which is important for salinity tolerance in *O. coarctata*. However, Gao et al. (2011) reported that osa-miR393a expression decreased under salt treatment, resulting in increased sensitivity to saline. In rice, miR393a is negatively regulated by salinity tolerance. Auxin response factor (ARF) belongs to the auxin signalling pathway family, which negatively regulates the growth and development of rice plants. Oco-miR044-3p targets the K^+^ antiporter gene, which is responsible for the conservation of Na^+^ and K^+^ homeostasis in *O. coarctica*. The oco-miR044-3p is also an important gene regulator of the survival of rice plants under saline conditions. Moreover, the sulfate transporter gene was predicted to be responsible for salinity tolerance in *O. coractata*. In rice seedlings, miR169, miR319a.2, miR408-5p, miR397, miR528, miR827, and miR1425 have been found to be involved in hydrogen peroxide-mediated responses (Bonnet et al., 2004) (Table 1). Some miRNAs were associated with direct stress responses and downstream signalling processes (Figure 2). For example, oco-miR020-3p, oco-miR026-3p, and oso-miR396c target downstream signalling pathway genes, such as phosphoinositide 3-kinase and leucine-rich repeat family proteins (Mondal et al., 2015).

Other miRNAs, such as miR167, miR319, miR812q, and miR1425, which are known as cold-stress-responsive miRNAs, regulate target genes during cold conditions (Jeong et al., 2011). On the other hand, osmiR397 is a high-temperature stress-responsive miRNA that regulates L-ascorbate oxidase expression in rice during heat stress (Sailaja et al., 2014). During microbial infection in rice, there is a fluctuation in miRNA expression, indicating that miRNAs are also involved in plant responses to pathogens. *Magnaporthe oryzae* is the causative agent of rice blast disease in *O. sativa*. Zhang et al. (2018) compared the expression of *miR319* in mock- and *Magnaporthe oryzae*-treated rice plants. They found that *M. oryzae* strain Guy11 induces the expression of miR319, which represses the target gene *osTCP21*. Moreover, *osa-miR398b* overexpression increases resistance to blast disease (Yang et al., 2012). A recent report states that there is a synergistic effect between nutrient supply and bacterial infection in *O. sativa*. For example, limiting the nitrogen source improves resistance to bacterial blight disease caused by *Xanthomonas oryzae* (Tran et al., 2018).

### Wheat

Wheat (*Triticum aestivum* L.) is an extensively cultivated crop plant consumed by humans worldwide, providing 25% of the calories per day. The miRBase v. 21 maintained 116 miRNA sequences belonging to 30 miRNA families in wheat. Wei et al. (2009) identified 58 wheat miRNAs (from leaves, stems, roots, and spikes) by sequencing, which belong to 43 miRNA families. Moreover, 32 miRNAs and their respective target genes were identified in wheat seedlings through degradome library construction by Li et al. (2012). Recently, 323 novel miRNAs and their respective target genes were identified in wheat, which are involved in grain development (Li et al., 2015). Similar to other crop plants, microRNAs have significant functions in the regulation of genes related to responses to biotic and abiotic stresses. A comprehensive analysis revealed the presence of 153 microRNAs (miRNAs) in wheat, which exhibit associations with both biotic and abiotic stress responses (Figure 2).

Fungal infection with *Blumeria graminis* f. sp. *triticum* (Bgt) in wheat results in the downregulation of miRNAs, such as miR156, miR159, miR164, miR171, and miR396, and the upregulation of miRNAs, such as miR393, miR444, and miR827 (Sun et al., 2014). *Fusarium culmorum* is a fungal pathogen that mostly affects small-grain cereals, such as wheat and barley, and is recognised as a significant contributor to biotic stress in these crops. According to Xin et al. (2010), the expression levels of three microRNAs, namely, gma-miR5783, gma-miR171, and ath-miR2933, are significantly upregulated in response to *Fusarium culmorum* infection. These microRNAs play a crucial role in providing protection against the fungal infection. Plant cells are subject to significant harm due to many environmental conditions, including salinity, heavy metals, UV radiation, and drought. This damage occurs as a result of the buildup of superoxide radicals, hydrogen peroxide, and hydroxyl radicals. The involvement of miR398 in the plant’s abiotic stress response has been observed in wheat. The two Cu-Zn SOD genes (CSD), cytosolic CSD1 and plastidic CSD2 were upregulated in miR398 expression. Under stress conditions, CSD1 and CSD2 mRNAs accumulate and downregulate the expression of miR398 (Inal et al., 2014).

Among environmental stresses, drought is considered the dominant abiotic stress, which occurs due to a shortage of rainfall or efficient evaporation of water from the soil. The expression of miR169g, miR171a, and miR393 was identified in response to dehydration, whereas miR169 accumulation was found to regulate the expression of C-repeat/dehydration-responsive element binding factor (CBF/DREB) TFs (Inal et al., 2014). Similar results were obtained for emmer wheat (*T. turgidum* ssp. *dicoccoides*), during drought conditions, 13 miRNAs like such as miR156, miR166, miR171, miR396, miR398, miR474, miR528, miR894, miR896, miR1432, miR1450, miR 1881, and miR 1867, were differentially regulated (Sunkar and Zhu, 2004). Under cold stress, upregulation of miR408, miR169, miR396, miR172, miR393, miR397, miR165/166, and other miRNAs such as miR156, miR157, miR159, miR164, miR319, miR394, and miR398 has been identified in *Triticum turgidum* ssp. *durum* (Kantar et al., 2011). In addition, microRNAs such as miR156, miR159, miR164, miR167a, miR171, and miR395 also exhibit differential expression patterns during ultraviolet-BUV-B (280–320 nm) stress in wheat (Zhang et al., 2009). Wheat is susceptible to stripe rust when grown in cool environments (Table 1). miRNAs such as miR167, miR171, miR444, miRl129, and miRl138 develop resistance against stripe rust (Gupta and Huang, 2014). Recently, abiotic stress response miRNAs, such as Ta-miR1122, Ta-miR1117, Ta-miR1113, and Ta-miR113 were identified in wheat (Figure 2) (Li et al., 2015).

### Maize

Maize (*Zea mays* L.) is the world’s second most cultivated crop variety after rice. Maize is primarily used as a food source by both humans and animals. In addition to being a food source, it is a major constituent of ethanol production. Maize is considered a model plant to study plant development and evolution, as well as to understand the regulatory functions of miRNAs (Wang et al., 2013). In maize, the secondary structure of the precursor was predicted based on the phylogenetic conservation of 21 nucleotides miRNA (Bothast and Schlicher, 2005). Moreover, the expression of different miRNAs varies with variations in the developmental stages of maize. After pollination for 10 days, miR169 expression was lower in kernels (Ambros et al., 2003). miRNAs, such as miRNA156, miR160, and miR169, are expressed during maize development in seedlings (Djami-Tchatchou et al., 2017). However, miR167 is expressed in both developing kernels and seedlings. miR156 targets TC294022 and TC280157, which encode the proteins involved in early flower development (Wang et al., 2015). The expression of miRNAs is genotype-specific, which has been proven by the analysis of miRNAs from the same tissues of different genotypes. For example, a higher level of miR166 expression was observed in the kernels of the inbred line H99 than in the B73 and B733H99 F1 hybrids. However, similar miR167 expression levels have been observed in both seedlings and kernels in inbred lines, such as H99 and B73 (Thiebaut et al., 2012). In maize, Sheng et al. (2015) have identified 29 miRNAs using high-throughput sequencing of 23 miRNAs as potential targets (Figure 2).

Like other plants, in maize, miRNAs are crucial in abiotic and biotic stress responses (Ding et al., 2011) such as low nitrate (Qin et al., 2011), phosphorous (Xu et al., 2011) and salt stress (Zhang et al., 2012). Drought is the primary factor limiting the growth and productivity of maize. Recently, high-throughput sequencing and bioinformatics tools have been used to identify novel miRNAs and their respective targets in maize (Thiebaut et al., 2014) (Table 1). Sheng et al. (2015) reported 124 conserved and 68 novel miRNAs that are related to drought stress responsiveness in maize. miR396, miR167 and miR169 are the key regulators of abiotic stresses in maize, which target Nzma- MIR396d, zma-MIR167h and small-MIR169a respectively (Sheng et al., 2015). The overexpression of miR172 targets the gene Glossy15, which is necessary for the expression of juvenile epidermal traits that affect flowering time in maize (Liu et al., 2014). miR395 regulates sulfur assimilation and translocation by controlling mRNA levels of ATP sulfurylase and low-affinity sulfur transporters (Lauter et al., 2005). Similarly, miRNAs such as miR398 and miR408 are copper-regulated miRNA (Cu-miRNA). Furthermore, miR397 and miR528 are also categorized under Cu-miRNA based on their function in the regulation of the protein to participate in copper homeostasis (Kawashima et al., 2011).

### Cotton

Cotton (*Gossypium hirsutum* L) is a fiber-producing crop, that is, considered to be the most economically significant crop variety (Zhang et al., 2014). Cotton plants are considered model plants for cell wall and cellulose biosynthesis studies. The genus *Gossypium* is a broad family comprising approximately 50 species that originated mainly in Australia and Latin America. Among them, only four species, cotton (*G. hirsutum*), island cotton (*G. barbadense*), African cotton (*G. herbaceum*), and Asiatic cotton (*G. arboreum*), are domesticated species that produce beneficial spinnable fibers, and the development of cotton fiber cells is composed of four stages: initiation, elongation, secondary cell wall synthesis, and maturation (Haigler et al., 2012). miRNAs play a crucial role in the development of cotton fibers. miR160d accumulates in fibers and fiber-bearing ovules. It targets ARF10, and its expression is higher during fiber initiation and development (Figure 2).

miR167b targets another auxin response factor, ARF8 (Wang et al., 2013). Among the different developmental stages of cotton fibers, the expression of miR167b was higher during the elongation stage. The target gene of miR447a is HSC70, which is involved in fiber development. Moreover, the expression of some miRNAs varies at each stage of fiber development. For example, miR394a shows diverse expression patterns at every stage of fiber development. The high expression level of miR394a was specifically responsible for rapid fiber elongation (Table 1; Figure 2). Squamosa promoter-binding-like protein 9 (SPL9) is crucial for floral organ trichome formation, fiber initiation, and elongation, and is targeted by miR156 (Wang et al., 2017). Cellulose synthase A catalytic subunit 8 (CESA8) and subunit 9 (CESA9) are targeted by miRn38, miRn65, and miRn68. The expression of these miRNAs differed in various tissues and was expressed at lower levels during elongation of the fiber and biosynthesis of the secondary wall. The transition from vegetative to reproductive growth is regulated by miR172 and miR156 (Gou et al., 2011). For example, miR156 downregulation enhances the expression of SPL9, which leads to miR172 upregulation. Certain miRNAs are co-regulated during fiber initiation and development. For example, miR156b was expressed at a lower level during fiber initiation and at a higher-level during elongation, whereas miR172e was upregulated during fiber initiation and at a lower level during elongation (Figure 2).

Wang (2014) identified 73 miRNAs that belong to 49 families from Asiatic cotton. Most miRNAs are involved in plant growth, development, and environmental stress responses. For example, miR172 is involved in floral development (Wang et al., 2015) and miR399 is responsible for environmental stress (Toetia and Tang, 2015). Hence, it can be easily grown on dry land with few cultivation practices. The miRNAs responsible for the regulation of the salt stress response in cotton have also been reported. For example, miRNVL5 regulates the expression of salt-tolerant genes, *G. hirsutum* cysteine/histidine-rich C1 (GhCHR) domain family protein, in cotton under salt stress and is considered a positive regulator of plant salt stress tolerance. Evidence has shown that Asiatic cotton is resistant to several pests, such as bollworms, aphids, leafhoppers, and microbes, such as fungal and viral infections (Yin et al., 2017). Asiatic cotton is inherently tolerant to abiotic stresses such as drought and salinity (Akhtar et al., 2010). miR2948 targets genes encoding sucrose synthase and glucose-methanol-choline oxidoreductase, which are responsible for fiber initiation and elongation (Liu et al., 2006). miR2950 targets gibberellin 3-hydroxylase 1, which controls fiber cell development. It also targets tubby bipartite transcription factor (*TUB*), which is highly expressed in elongating fiber cells (Ruan et al., 2003; Tahir et al., 2011). However, Wang et al. (2017) reported that a lower level of *TUB* expression in fiber elongation showed that the plant metabolic process influences the expression of *TUB*. The expression of miR2950a was poor in immature ovules and higher in the fiber elongation stage. The only miRNA expressed in different fiber development stages is miR447a which targets numerous genes and transcription factors such as actin 7 (ACT7), *annexin D2* (Ann2), *bHLH093*, *CPC, GL3, MYB16, MYB88* and *TUB1* (Table 1; Figure 2).

### Coffee

Coffee (*Coffea arabica*) and *Coffea canephora* L.) is a commercial non-alcoholic beverage crop that contains several beneficial metabolites, such as caffeine, polyphenols, chlorogenic acid, and caffeic acid (Mlotshwa et al., 2006). The genus *Coffea* consists of 100 species; however, *C*. *arabica* and *C*. *canephora* are the two most economically significant and extensively cultivated coffee species. It has been suggested that coffee consumption diminishes the risk of Parkinson’s disease, Alzheimer’s disease, heart disease, type 2 diabetes mellitus, gout, and liver cirrhosis (Cheng et al., 2011). Moreover, coffee development and production are also affected by diverse environmental conditions, such as drought, salinity, heat, and fungal infection of coffee leaves (Del Terra et al., 2013). MicroRNA studies provide necessary information regarding the development and function of target genes in coffee plants. However, the prediction of target genes of miRNAs has been functionally annotated, depending on the molecular function, biological component, and process. Compared to other plant species, studies on the functional role of miRNAs in coffee are lacking. To date, miRNAs have not been deposited in miRBasev.21 for the genus *Coffea*.

The two distinct sources expressed sequence tags (EST) and genome survey sequences (GSS), contain information on the complete plant genome of coffee (Vidala et al., 2010). NCBI has deposited 17, 4275 ESTs and 3757 GSS of *C. arabica* (Lima et al., 2013). ESTs and GSS provide crucial information for predicting novel miRNAs and their corresponding genes in *C. arabica*, which is considered to be of economic importance. Recently, 20 feasible miRNAs from 13 miRNA families (car-miR393, carmiR393b, car-miR393d, car-miR393b-3p, car-miR390a, car-miR390b, car-miR390d-3p, car-miR390e, car-miR482d, car-miR2118e, carmiR397b, car-miR533, car-miR854d, car-miR426, car-miR1879, carmiR5809, car-miR414, car-miR1134, car-miR1110, and car-miR5122) were successfully identified in *C. arabica* by ESTs and GSSs (Akter et al., 2014) (Table 1; Figure 2). miR393 has been identified in coffee, which targets the genes that encode transport inhibitors, such as proteins, DNA-binding proteins, TFs, and GRR1-proteins, which are involved in chitin, cold, salt, and water deprivation stress responses, respectively (Dezulian et al., 2006) (Figure 2).

Interestingly, car-miR397b targets the gene encoding for laccase 2 (LAC2) which is involved in Copper (Cu) homeostasis and lignin metabolism. Under heat stress, increased monolignol accumulation was reported in the leaves of *C. arabica* (Del Terra et al., 2013). In coffee, a single miRNA can have multiple target genes. For example, car-miR1134 targets mRNA responsible for oxygen binding, water channel activity, and metal ion binding. Similarly, the miR393 family targets auxin signalling genes, such as transport inhibitor response 1 (*TIR1*), *AFB2*, auxin signalling F-box3 (*AFB3*), and glucose repression-resistant (*GRR1*) - protein 1. CarmiR2118e and car-miR482d target mRNA encoding polygalacturonase, which is responsible for carbohydrate metabolism. Biotic factors also affect the development and economic traits of coffee. For example, coffee leaf rust is a primary disease of coffee plants caused by the fungus, *Hemileia vastatrix* (Del Terra et al., 2013; Rebijith et al., 2013). However, car-miR428d and car-miR2118e are responsible for the defense mechanisms in coffee. Car-miR428d and car-miR2118e target the gene coding for the disease-resistant protein, which contains nucleotide-binding ARC (Devi et al., 2016). In their study, Loss-Morais et al. (2014) revealed that miR8697 exhibits a wide range of anticipated targets, including nucleoside diphosphate kinase group I (NDPK-I)-like, NADH dehydrogenase, ubiquitin domain of GABA-receptor-associated protein, and drought-induced 19 proteins (Di19). miR8558 belongs to the miR482 family, which possesses target genes responsible for plant innate immune receptors, such as nucleotide-binding site-leucine-rich repeats (Zhu et al., 2013).

### Tea

Tea [*Camellia sinensis* (L) Kuntze] is the world’s most important non-alcoholic beverage crop and is primarily grown in China, India, and Southern Asia. Tea contains numerous bioactive secondary metabolites such as polyphenols, caffeine, and theanine (Ramkumar et al., 2012). These compounds possess various health benefits, including anti-cancer, anti-microbial, and immune-regulatory effects, and can reduce the risk of cardiovascular and neurological diseases (Samynathan et al., 2023). The miRNA content in tea varied depending on the tea variety and processing time. For example, csn-miR164 content varied among distinct varieties of commercial green tea whereas, the content of miR329 varied according to tea processing (Hou et al., 2017). Sun et al. (2014) predicted the targets of 97 miRNAs which regulated 216 genes in tea plants. Among these, 19 miRNAs belonging to 16 miRNA families targeted 26 genes responsible for post-transcriptional expression regulation. Multiple miRNA families regulate common targets in other plant species. For example, in 1005 different tea cultivars it is predicted that csn-miR160a regulates two ARF genes such as *ARF8* and *ARF6* which were homologous to *ARF18* in *O. sativa* (Po-Pu et al., 2007) and *Arabidopsis thaliana* (Liu et al., 2006). The levels of miRNA expression were found to be much greater in the initial leaves compared to the mature leaves. This observation suggests that these particular miRNAs have a significant impact on the process of catechin production. There is a strong correlation between csn-miR4380a and dihydroflavonol 4-reductase (DFR) expression in tea plants. In particular, csn-miR160a, csnmiR167a, and csn-miR396a played major roles in the development of tea leaves, as well as in the regulation of catechin biosynthesis (Figure 2). In contrast, miR2911 is involved in caffeine metabolism in tea (Sun et al., 2014).

In tea, miRNAs such as novel-miR1, csn-miR426 and csn-miR482 are playing an essential role in disease resistance (Jeyaraj et al., 2020). Jeyaraj et al. (2017) identified miRNAs that are responsible for insect defense in tea plants, and miRNAs such as csn-miR156, csn-miR172, and csn-miR319 are also associated with biotic stress responses in tea. Zhu et al. (2013) have identified 14 new miRNAs that target a total of 51 mRNAs, which are responsible for oxidation-reduction, stress responses and transportation. In addition, a total of 130 conserved miRNAs and 512 unique miRNAs have been identified, which play crucial roles in several biological processes such as transcription, signal transduction, stress response, and plant development and maintenance. The infestation of a herbivore (*Ectropis oblique*) in tea plants leads to the expression of over 150 miRNAs (Jeyaraj et al., 2017).

Drought is a major environmental stress in tea and decreases tea production by 14%–33% (Chen et al., 2022). In addition to drought, cold stress is an important factor that affects the productivity of tea manufacturing (Li et al., 2020). Guo et al. (2017) reported that conserved miRNAs, such as miR156, miR166a, and miR398, are responsible for drought stress resistance in tea plants. Zhang et al. (2017) identified 18 conserved and 14 novel miRNAs in tea plants in response to cold stress, using a microarray technique. Moreover, they observed that 31 miRNAs were upregulated and 43 miRNAs were downregulated in cold-tolerant plants, and 46 miRNAs were upregulated and 45 miRNAs were downregulated in cold-sensitive tea plants (Zhang et al., 2014) (Table 1; Figure 2).

### Legumes and oil crops

Cereals and oil crops are economically viable crops cultivated in numerous countries worldwide. In their study, Sha et al. (2012) employed high-throughput short RNA sequencing and whole-genome mining techniques to detect potential microRNAs in *Glycine* max under conditions of phosphorus (P) deficiency. The researchers made the observation that the roots exhibited notable alterations in the expression of 27 established, 16 universally conserved, and 12 newly discovered miRNAs when subjected to phosphorus shortage. Similarly, the shoots also exhibited significant disparities in the expression levels of 34 established, 14 conserved, and 7 distinct microRNAs in response to phosphorus (P) deficiency. In their study, Turner et al. (2012) documented the presence of four previously unidentified microRNAs, namely, gma-new-miR4416a, gma-new-miR4416b, gma-new-mi13587, and gma-new-miR50841, which exhibited significant levels of expression in *G. max*. In their study, Ho et al. (2017) successfully identified several miRNAs, including miR156, miR160, miR166, miR167, miR168, miR172, miR396, miR528, and miR535, within the floral meristems of oil palm. These miRNAs probably work in tandem with their mRNA targets to regulate the development of early floral organs.

Gupta et al. (2017) revealed that *ARF10, ARF16*, and *ARF17* gene expression improved significantly during early embryogenesis in the miR160 floral organs in carpels (foc) mutant of soybean. The microRNA miR167 demonstrates a prominent activity throughout the later stages of embryogenesis, specifically targeting the genes ARF6 and ARF8. The investigation of the chickpea transcriptome unveiled the presence of hitherto unidentified miRNAs, namely, Car-novmiR61, Car-novmiR1, and Car-novmiR218. These miRNAs have been implicated in the regulation of seed size and weight, as reported by Garg et al. (2017). Ding et al. (2018) conducted a study to examine the oil levels, specifically linoleic acid (42%) and linolenic acids (39%), in the sea buckthorn plant. Through deep sequencing, the researchers identified a total of 137 known miRNAs from 27 families, as well as 264 previously unidentified miRNAs. The present investigation suggests that many miRNAs, namely, miR164d-ARF2, miR168b-Δ9D, novelmiRNA-108-ACC, novelmiRNA-23-GPD1, novelmiRNA-58-DGAT1, and novelmiRNA-191-DGAT2, may play a role in the control of seed size and lipid production in sea buckthorn.

Zhao et al. (2020) explored the molecular and functional regulation of miRNAs during walnut (*Juglans regia*) seed development. A total of sixteen modules involved in the regulation of miRNA-mRNA interactions have been identified, specifically encompassing jre-miRn105, jre-miRn434, jre-miR477d, and jre-miR156a. These modules have been found to be related with the processes of fatty acid generation and oil production. Jiang et al. (2018) investigated the miRNA profiling in the male sterile lines of *Brassica napus* and they identified 44 new conserved miRNAs, 27 unique miRNAs, and 46 known miRNAs. Moreover, MiR159 was selected as a candidate for analysis of its role in male sterility and reproductive development from the differentially expressed miRNAs. In their study, Khemka et al. (2021) reported differential expression of the miR156 family in two chickpea cultivars. Specifically, they observed an antagonistic expression pattern at the target of the SPL genes. Salgado et al. (2022) investigated the preliminary response of an oil palm (*Elaeis guineensis* Jacq.) crop to 2 weeks of water scarcity. Among the 81 miRNAs observed in this study, 29 were specific to the oil palm, and egu-miR28ds and egu-miR29ds were newly identified. Furthermore, it was observed that a total of 62 miRNAs exhibited differential expression under conditions of drought stress. Among these, five miRNAs (miR396e, miR159b, miR529b, egu-miR19sds, and egu-miR29ds) were found to be upregulated, while the other 57 miRNAs displayed downregulation. Notably, the upregulated miRNAs included miR396e, miR159b, miR529b, egu-miR19sds, and egu-miR29ds. In the context of water stress in oil palms, several transcription factors (TFs) including MYBs, homeodomain-containing (HOXs), and nuclear factor-Y (NF-Ys) have been recognised as potential target genes for microRNAs.

miRNAs such as miR159.2, miR393, and miR2118 in *Phaseolus vulgaris* were greatly induced under ABA administration, while the other miRNAs, namely, miRS1, miR1514, and miR2119, showed a slight response to ABA (Arenas-Huertero et al., 2009). The new miRNAs found in common beans, along with different legumes, have a role in the regulation of processes unique to legumes, such as adaptability to various environmental signals. According to Wang et al. (2013), pea plants presumably possess miRNAs miR319, miR393, miR397, and mi402, and their expression levels were increased in response to cold treatment. The study conducted by Li et al. (2010) aimed to assess the gene expression levels of six distinct families of novel miRNAs and their role in the process of soybean nodule formation. The results of their study indicate that miR482, miR1512, and miR1515 potentially have different and significant functions in the process of soybean nodulation. In their study, Yan et al. (2011) examined the functional implications of four microRNAs (miRNAs), namely, Gma-miR2606b, miR1514, TAG2382310, and Gma-miR4416. These miRNAs were shown to exhibit unique characteristics in relation to legumes, with Gma-miR2606b and miR1514 being peculiar to this plant family. Additionally, TAG2382310 and Gma-miR4416 were identified as miRNAs that are specifically associated with soybeans. Based on the findings of Kozomara et al. (2019), it has been shown that miRBasev.21 provides access to a total of 10 mature miRNAs for common bean (*P. vulgaris*), 790 for *Magnaporthe truncatula*, 756 for soybean, 365 for *L. japonicus*, and 790 for soybean. A comprehensive count of miRNAs has been conducted for many plant species, including *P. vulgaris*, *M. truncatula*, soybeans, *L. japonicus*, and soybeans. The miRBasev.21 database has shown a total of 790, 756, 365, 790, and 10 miRNAs for each individual species. Table 2 depicted miRNAs targets and their functions in cereals and other crops.

**TABLE 2:** microRNAs (miRNAs) targets and their functions in cereals and other crops.

Plants	miRNA—Upregulation	Functions	Target genes	References
Barley (*Hordeum vulgare*)	miR160	Auxin-mediated genes regulation	Auxin response factor (ARF)	Tombuloglu (2019)
	miR171	Regulation of seed germination	Scarecrow transcription factors (TFs)	Dou et al. (2021)
	miR166a, miR166′b, miR166c	Vascular differentiation, development of leaf and root formation	HD-Zip protein 8	Yadav et al. (2021)
	miR397a	Plant and flowering development	laccase precursor protein; transporter family protein	Huang et al. (2020)
Sorghum (*Sorghum bicolor* (L.) Moench)	miR156a, miR156b, miR156c-f, miR156g-h, miR529, novel-sbi-miR-119, novel-sbi-miR-383, novel-sbi-miR-329, & a novel-sbi-miR-350	Stress in response to the drought	Squamosa promoter binding protein (SBP) gene	Katiyar et al. (2015)
	miR529	Stress in response to the drought	Protease and ubiquitin-related genes	
	miR398	Stress in response to the drought	Cu/Zn SOD, selenium-binding protein, and cytochrome C	
*Pennisetum glaucum* (L.) R. Br. (Pearl millet)	miR155	Stress in response to the drought	Cu-Zn superoxide dismutase, *eIF-4A*	Chakraborty et al. (2020)
	miR399, KN1		*NCED1, LOV,* aspartate aminotransferase	
*Setaria italica* (L.) P. Beauv. (Foxtail millet)	miR160	Stress in response to the drought	*WRKY* and *ARF*	
	miR165 Carboxylesterase		*WRKY, ARF, NDR1/HIN1*	
*Glycine* max L.)	777 miRNAs	Metabolism of phosphorus and stress response to biological regulation	*ERF, ARF, MYB,* and *NAC*	Liu et al. (2020)
*Glycine* max L.)	miR169	Reduce the stress response transcript	*AtNFYA1* and *AtNFYA5*	Yu et al. (2019)
*Camellia oleifera*	ath-miR858b–	Seed oil biosynthesis	*MYB82/MYB3/MYB44*	Wu et al. (2021)
	csi-miR166e-	Formation and accumulation of oleic acid	*5p–S-ACP-DES6*	
*Brassica napus*	bna-miR165a-5p	wax biosynthesis	BnaA06g40560D (*CYP96A2*)	Liu et al. (2019)
Pear (*Pyrus communis* L.)	Pyr-miR171	Improvement of shoot development	*PyrSCL6* and *PyrSCL22*	Jiang et al. (2018)
Cotton (*Gossypium herbaceum* L.)	miR414c	Improvement of primary root growth and biomass	*Iron SOD gene (GhFSD1)*	Wang et al. (2019)
	miR156	Stress response	*SBP/SPL TFs*	Wang et al. (2015)
	miR395	Salt and drought stresses	*APS1*	Wang et al. (2013)
*Ricinus communis* L	miR398	The control of cellular growth	*Cu–Zn/SOD*	Çelik and Akdas (2019)
Pear	Pyr-miR171	Enhancement of shoot development	PyrSCL6 and PyrSCL22	Jiang et al. (2018)
*Ricinus communis* L	miR398	The control of cellular growth	Cu–Zn/SOD	Çelik and Akdas (2019)
Coffee (*Coffea arabica* and *Coffea canephora*)	miR167	Development and stress response	Auxin response factor	Chaves et al. (2015)
	miR171	Development and metabolism	GRAS family TFs	
	miR159e	Stress response and development	Medium chain reductase/dehydrogenases	Loss-Morais et al. (2014); Chaves et al. (2015)
Tea (*Camellia sinensis.* (L). Kuntze)	csn-miR162a, csn- miR394a	Growth and development	*ARF5, NAC89, PAL, MYB75*, and *WRKY*	Jeyaraj et al. (2017)
	csn-miR169e, csn-miR399b_1ss21GA, csn-miR408-p3_2ss18GT19GT, csn-miR477g-p5, and PC-5p-80764_22	Abiotic and biotic stresses response		
	csn-miR828a, csn-miR858b, csn-miR858a, csn-miR6300, csn-miR164a, and csn-miR169)	The positive regulator of the response to abiotic and biotic stress	*MYB, NAC*, and *NF-YA*	Jeyaraj et al. (2019)
	novel-miR1, csn- miR426 and csn-miR482	Disease resistant	*WRKY* TF, LRR protein kinase, Ser/Thr-kinase, and LRR receptor	Guo et al. (2017)

## Regulation of seed miRNA

Seed growth and germination are two important developmental stages in the plant life cycle. As in most flowering plants, seed germination and maturation are isolated by a time of quiescence, referred to as dormancy. miRNAs effectively regulate gene expression during seed growth and germination (Das et al., 2015). MicroRNAs and other small RNAs are known to exert significant influence on the processes of seed germination and dormancy. miRNAs are active in both sustaining and breaking dormancy to facilitate the development of the embryo to the seedling level via seed germination (Zhang et al., 2017). In plants, the differentially expressed miRNAs miR417, miR164, miR158, miR160, miR156, miR167, miR395, miR159, miR165/166, miR402, and miR172 are known to monitor all seed germination and dormancy activators and repressors (Das et al., 2015). MiR164 was primarily expressed at the development and post-germination stages of seeds; miR396, miR172, and miR393 were expressed selectively in the embryo 5 days after germination (Bai et al., 2017). It is anticipated that a diverse range of microRNAs present in somatic embryos of Norway spruce contribute to the establishment and maintenance of epigenetic memory associated with seed development (Yakovlev and Fossdal, 2017). In maize seed embryos, 12 miRNA families were identified, including miR159, miR397, miR319, miR167, miR393, miR394, miR172, miR169, miR156, miR168, miR164, and miR166, which have been shown to be differentially downregulated, whereas miR398, miR408, miR528, and miR529, have been upregulated during the seed germination stage (Li et al., 2013). Gu et al. (2013) investigated two developmental stages of maize endosperm through small RNA libraries and found that 18 new miRNAs involved in endosperm production have been discovered and classified into 11 families. A comprehensive investigation of miRNAs was conducted on wheat tissues, encompassing the entire genome. This search resulted in the identification of 323 previously unreported miRNAs, which can be classified into 276 distinct families. Additionally, the target genes associated with these miRNAs were also determined. According to Sun et al. (2014), an increased abundance of these miRNAs was seen in grains, suggesting their significant involvement in the process of grain synthesis. Miura et al. (2010) conducted a study on rice plants and found that miR156 has a suppressive effect on the gene *OsSPL14*. This gene plays a crucial role in regulating the growth of both vegetative and reproductive organs, as well as determining the optimal architecture of the plant. Specifically, the suppression of *OsSPL14* by miR156 leads to an increase in tiller production, a reduction in plant height, and an enhancement in culm diameter, panicle branches, and grain number. In rice, miR398 undergoes some modifications to improve grain number, panicle length, and grain size (Zhang et al., 2017). Additionally, miR164b is highly expressed in rice and shows a good architecture of the plant body with lengthy panicles, high grain number, and good yield (Jiang et al., 2018). In a subsequent investigation, Zhao et al. (2019) found a correlation between the expression of miR1432 in rice and its impact on grain size and yield. Additionally, the researchers observed that OsACOT has a role in the production of fatty acid chains. According to Zhang et al. (2013), the augmentation of rice yield was achieved through the upregulation of OsmiR397, a microRNA, which led to enhancements in grain size and the promotion of panicle branching in rice.

The use of miRNA-based genetic engineering in *Camellia* could facilitate molecular breeding of new ornamental varieties with desirable floral forms. In *Camellia*, Yin et al. (2016) investigated the functional effects of carpel-specific miRNA–target pairs for the annotation of fruit development and the miRNA160–ARF were correlated with carpel formation. Yin et al. (2016) exploited high-throughput small RNA sequencing they identified 175 mature and precursor sequence miRNAs that are closely related to organs developed by *Camellia azalea*. In this study, miRNA167, miRNA156, and miRNA159 were identified to be involved in floral organ growth. Differential miRNA expression patterns were observed in *C. meiocarpa* and *C. oleifera* across varying environmental conditions, notably in the context of desiccated seeds. This observation implies that miRNAs might exert a significant influence on lipid metabolism throughout the process of natural seed desiccation. In their study, Feng et al. (2017) conducted an investigation utilising GO and KEGG role annotation. Their findings revealed that a total of 23 miRNAs were identified as regulators of lipid metabolism, specifically involved in the regulation of fatty acid biosynthesis, accumulation, and catabolism during the natural drying process of seeds. These miRNAs were discovered to affect a total of 131 target genes. In the study conducted by Jeyaraj et al. (2019), it was observed that infection of tea plants (*C. sinensis* L. var. shuchazao) with *Colletotrichum gloeosporioides* resulted in the downregulation of three specific microRNAs (csn-miR5368-p5 2ss15TC18CT, PC-3p-106557 16, and PC-3p-70583 26). These microRNAs were found to target the Ser/Thr protein kinase. Additionally, the LRR receptor-like Ser/Thr protein kinase was targeted by csn-miR156a-p3 1ss10TA, leading to its downregulation. Wu et al. (2020) identified twenty-three miRNA-mRNA regulatory modules associated with seed size regulation in *Camellia oleifera*. Among these modules, a negative regulatory module involving hpe-miR162a L-2- ARF19 was shown to be involved in the early stages of seed growth.

## Summary and future perspectives

MicroRNAs (miRNAs) are known to exert significant influence on the regulation of genes associated with several aspects of plant growth and development. Moreover, miRNAs participate in the environmental stress response, which influences agronomic traits in crop varieties. Maize and sorghum are evolutionarily related to rice, and maize miRNA genes are orthologs of the rice genome compared to sorghum. Crop production is primarily affected by salinity, which is considered a significant abiotic stressor (Yadav et al., 2021). The miRNAs responsible for the regulation of salinity stress establish an adaptive mechanism for plants to that particular environment. Ding et al. (2009) reported that there are 98 miRNAs belonging to 27 families exhibited differential expression in maize roots treated with salt stress. In the context of salt stress in maize roots, it was shown that miR156, miR164, miR167, and miR396 exhibited downregulation, while miR162, miR168, and miR395 displayed upregulation. The miR482/miR2118 superfamily plays a role in the activation of defence mechanisms in different plant species through the regulation of NBS-leucine-rich repeat (NBS-LRR) genes (Arenas-Huertero et al., 2009). MiRNAs are potential gene regulators in plants. According to Jiang et al. (2018), miR164b exhibited a significant upregulation in rice plants, resulting in a well-developed plant structure characterised by elongated panicles, increased grain count, and enhanced overall yield. Jeyaraj et al. (2020) identified tea miRNAs like csn-miR156, csn-miR172 and csn-miR319 are insect defense-related tea plants miRNAs are also associated with biotic stress responses in tea. In their study, Wu et al. (2020) identified a total of twenty-three miRNA-mRNA regulatory modules that play a crucial role in the regulation of seed size in *C. oleifera*.

Numerous studies have demonstrated significant roles of miRNAs in the regulation of plant genomes. Understanding their functions and interactions with target genes provides valuable insights into improving stress tolerance in agronomic plants, which is crucial for sustainable agriculture and food security in a changing climate. The functions of miRNAs in the regulation of both abiotic and biotic stress responses in agronomic plants are summarized in this review, which is an excellent method for enhancing the growth and development of agronomic plants. The development of transgenic plants that express miRNA with the desired characteristics is ideal for improving the agronomic traits of the plant species. However, many agronomically important crops do not have complete or comprehensive miRNA profiling, which necessitates the development of complete miRNA profiling under normal and different a/biotic stresses. The provided information would be valuable for examining the potential functions of miRNAs in the context of plant development and their ability to withstand stress. In addition, the application of functional genomics methodologies will prove valuable in the elucidation of the specific roles attributed to individual microRNAs. Previous research on miRNA-mediated regulation of abiotic and biotic stress responses in agronomic crops has made remarkable progress, offering promising avenues for crop improvement. However, addressing the existing research gaps and challenges and embracing emerging technologies and interdisciplinary approaches will be essential to harness the full potential of miRNAs for sustainable and resilient agriculture in the face of increasing environmental stressors.
